# Traditional knowledge system in disaster risk reduction: Exploration, acknowledgement and proposition

**DOI:** 10.4102/jamba.v11i1.484

**Published:** 2019-06-04

**Authors:** Pribat Rai, Vimal Khawas

**Affiliations:** 1 Department of Geography, Sikkim University, Gangtok, India; 2 Department of Peace and Conflict Studies and Management, Sikkim University, Gangtok, India

**Keywords:** traditional knowledge, indigenous knowledge, scientific knowledge, disaster risk management, disaster risk reduction

## Abstract

The last 60 years have witnessed advanced technological innovation for disaster risk reduction (DRR) with the invention of high-resolution satellite imagery, digital cartography and modern engineering building techniques to high-yielding agricultural production. However, none have been highly satisfying in lessening the impact of disasters. The significant factor for the limited success of modern scientific society is that it views the world from a temporal perspective where humans are believed to be an active agent in modifying every natural possibility into opportunity. The very composite environmental system is simplified whilst extracting resources, resulting in resource depletion and environmental degradation, consequently opening the door for disaster. Technocratic science must recognise the need for a relational or holistic approach rather than believing in reductionist approaches alone whilst dealing with natural calamities. In this context, the knowledge of traditional societies is important to fill up the existing gaps created by the modern society. Traditional knowledge has different sets of ingredients to foster the development of the relational or holistic approach as it involves, interacts and interconnects humans, non-humans (animals and plants) and nature together, setting a perfect balance for sustainable development and DRR. It has vast undocumented observational data of changing natural phenomena, and in today’s scenario of climate change and uncertainty, it can create a path for reliable adaptation measures from climate-induced disasters. Thus, a holistic approach is needed for comprehensive DRR measures where both scientific and traditional knowledge systems can work together. The main purpose of this article was to explore the effective ingredients of traditional knowledge in DRR and how this age-old wisdom can be offered a hand to its integration into and collaboration with scientific research and management for DRR. To fulfil the objectives, a theoretical desk study approach was followed by identifying relevant studies, highlighting traditional knowledge in DRR from empirical and grey literatures, archive materials, biblical stories and so on. This research highlights some of the good practices of traditional knowledge in DRR and the possible path of collaboration of two knowledge systems in DRR.

## Introduction

Since the time of human evolution, humans had to face various natural calamities that even threatened their very existence on this planet. There are many ancient historical events where natural calamities nearly wiped out human civilisation. Theorists such as Jared Diamond (2005) and Dr Floyd McCoy (Cecil 2011) expressed that many of the great civilisations in history, such as the Mayan, the Minoan and the old Egyptian Empire, were ultimately brought to their knees not by their enemies but by the effects of floods, drought, famine, earthquake, volcanic eruption, tsunamis and other widespread disasters (Coppola 2011; Fagan 1999).

Ancient and historical stories have detailed recordings about the great natural disasters, their causes and their impacts upon mankind. For example, the extinction of the Tang Dynasty in China is believed to be a result of the yearly shift of the monsoon, which resulted in mass crop failure during the 8th and 9th centuries (Coppola 2011). However, many ancient and historical stories have also highlighted the response of humans during the said disastrous events. For example, the biblical story of Noah’s ark has depicted the very image of a huge flood on the earth’s surface and has given the account of early prediction, preparedness and mitigation strategies as presented in the following statement:

Make thee an ark of gopher wood. Thou shall make rooms in the ark, and shall pitch it inside and outside with pitch (Genesis 6:14). And this is how thou shall make it: The length of the ark three hundred cubits, the breadth of it fifty cubits, and the height of it thirty cubits (Genesis 6:15). And of every living thing of all flesh, two of every sort thou shall bring into the ark, to keep them alive with thee. They shall be male and female. (Gn 6:19)

The above quotation shows that the protagonist in the story of Noah’s ark attempts to mitigate the impact on the planet’s biodiversity by collecting two of each species and placing them within the safety of the ark (Coppola 2011). Materials and methods used to build the ark are also well recorded in the Bible (Ullendorff 1954). Although the story of Noah is normally considered as a myth because of the lack of physical evidence of a global flood (Radford 2014), the event still highlights the prediction and mitigation phase of flood disaster.

Similarly, the ancient flood mitigation strategic story has also been found in the ancient civilisation of Egypt under the reign of Amenemhet III, 1817–1722 BC (Coppola 2011). Using a system of more than 200 water wheels, the first river control project in history was established by the Pharaoh to divert the annual floodwaters of the Nile River into Lake Moeris. This helped Egyptians to reclaim over 153 000 acres of fertile land (Coppola 2011).

Traditional knowledge holds the element of those ancient stories, myths and folklores which, according to the modern scientific worldview, does not have any rational explanation because the scientific knowledge system does not believe in subjective reality. Measurement, experimentation and verification are one of the prime steps of scientific modern knowledge. However, modern scientific society forgets that those ancient stories and folklores have been developed through centuries-old experiences with the natural environment.

Gad-el-Hak (2008) postulates that nature is supreme in governing any natural law and natural laws govern the evolution of any disaster. Although humans advanced themselves from primitive civilisations to modern scientific civilisations, they still lack the understanding of nature’s activities or ability to predict its outcome. Modern science, for example, does not have precise laws in predicting earthquake hazards, thus making earthquake prediction more or less a black art (Gad-el-Hak 2008). The unpredictability of nature’s work still shows mankind that nature is the actual boss or dominant player. The United States Geological Survey (n.d.) has also stated the following:

No. Neither the USGS [United States Geological Survey] nor any other scientists have ever predicted a major earthquake. We do not know how, and we do not expect to know how any time in the foreseeable future. (n.p.)

Even the institutional framework for disaster risk reduction (DRR) is roughly 50–60 years old. For example, the United Nations General Assembly formally began to recognise the need for emergency assistance in case of any disaster only from the 1960s onwards when it passed resolution 2034 in 1965 (UNISDR n.d). Since then a number of DRR frameworks have emerged in global circles, such as the Yokohama Strategy in 1994, Hyogo Framework 2005–2015 and the recent Sendai Framework 2015–2030 for DRR (UNISDR n.d). However, our lack of understanding of nature’s outcomes and activities has failed to decrease the impact of disaster.

On the contrary, the indigenous and local communities worldwide have prepared, operated, acted and responded to disasters using their indigenous methods and passed them on from one generation to the next, even before the invention of high technology-based early warning systems, or standard operating procedures for response (UNISDR 2008). The present article attempts to explore traditional methods and practices in DRR, which have been highly acknowledged for being an effective evidential measure in reducing disaster risk but have been ignored by modern conventional knowledge because of the absence of scientific explanation or validity. The article also highlights the risk of the dominance of one form of knowledge in DRR and supports the significance of integrated knowledge bases to build a safer world. Thus, the article proposes collaboration or integration between modern scientific knowledge and traditional knowledge to develop a holistic approach towards DRR.

### Problem statement

The modern world usually ignores the traditional knowledge of indigenous and local communities treating it as mystic and instead focuses largely on technocratic solutions, denying the wider historical and social dimensions of environmental hazards (Mercer 2007). Even the current practices in disaster management and development too are shifting towards a modern thought, ignoring the old traditional values and culture.

Despite advancement in knowledge and technology today, such as satellite coverage and surveillance techniques, vulnerability to and risks from natural hazards have been increasing in developing and developed countries (Deken 2007). The 1995 Kobe earthquake in Japan killed more than 6000 people (Katayama 2004), the 2005 Hurricane Katrina in the United States killed about 1833 people (Zimmermann 2015), the 2011 Tohoku earthquake in Japan killed 15 894 people (Oskin 2017) and the 2017 Hurricane Harvey in the United States killed 88 people (Afiune 2017). This raises the question about the capability and sustainability of the so-called modern civilisations and their advanced technology in reducing disaster risks in future. Are we moving towards the brighter side of human civilisation or are we still not conscious about what we must do?

The concept of sustainable development and DRR cannot go much further with the guidance of the technocratic paradigm alone where the physical space and humans are isolated from each other. Traditional knowledge, as alluded by Mercer (2007), holds large amounts of information about the natural world because of its centuries-old experiences and observation of changing natural phenomena. Thus, traditional knowledge can complement current modern practices in the period of climate change and climate unpredictability. The application of traditional knowledge of indigenous and local communities in DRR needs to be explored to bring out the necessary ways in bridging the gap in DRR and to pave the future way for developing an integrated framework for DRR.

### Conceptual framework

Traditional knowledge refers to the undocumented knowledge or oral knowledge which has been passed down from generation to generation to a particular cultural community, in the form of stories, songs, folklore, proverbs, cultural beliefs, rituals, community laws, local languages, culinary recipes and agricultural practices (Connor 2003). However, the word ‘traditional’ normally implies the static nature of knowledge by many academicians and researchers. For example, D. Michael Warren, director of the Center for Indigenous Knowledge for Agriculture and Rural Development, Iowa State University, denotes the word ‘traditional’ as the 19th-century attitude of simple, savage and static (Warren 1995 in Berkes, Colding & Folke 2000). For this reason, some scholars favour the less value-laden term ‘indigenous knowledge’ (Berkes et al. 2000) which highlights the autochthonous nature of the internal origin and culturally integrated knowledge (Antweiler 1998) of native people, thus making thing easier in denoting a particular group of people (Nakashima & Roue 2002). However, the above concept of ‘indigenous knowledge’ often ignores the knowledge from local communities who are not officially recognised as indigenous.

In most of the developing regions of Asia and Africa it is not wise enough to use the word ‘indigenous’ and any attempt to designate one group as indigenous but not another may provoke confusion (Nakashima & Roue 2002). Another example comes from central Tanzania where paddy rice has been grown only since the 1930s, when it was introduced by Asian immigrants to the area (Shaka, Ngailo & Wickama 1996). It is now widely grown by African farmers, all of whom consider rice cultivation to be an indigenous activity (Shaka et al. 1996). This example further raises the issue about what actually constitutes ‘indigenous’, and how much it can be a contested term.

Distinctions between indigenous knowledge and traditional knowledge exist on the basis of academic discipline, context or language (Kelman, Mercer & Gaillard 2012), and they are not necessarily accepted as synonymous. The concept has a sufficient overlap, which allows them to be used interchangeably (Ryser 2012). However, the term ‘traditional knowledge’ would appear to be a more encompassing one (Busingye & Keim 2009) and normally would refer to a more generalised expression of knowledge associating both the indigenous as well as the local people with time-honoured ideas and practices (Ryser 2012).

The concept of being outdated or oldness is always mistaken whilst defining traditional knowledge. Traditional knowledge is based on existing knowledge (Haugen 2005) tested by trial and error and is transmitted to future generations orally or by shared practical experiences (Berkes et al. 2000). Dutfield (n.d.) rightly states that ‘traditional innovation’ is the ability of traditional knowledge to change with the change in circumstances of the relevant people, groups, community or region (Le Gall 2012). It can be held by one person, many people or everyone belonging to a local people or an indigenous community (Haugen 2005). Thus, the term ‘traditional knowledge’ brings together the knowledge of indigenous and local people who are not officially recognised as indigenous. This article focuses more on the conceptual view of traditional knowledge of indigenous and local communities in DRR.

## Methodology

This study applied a theoretical desk study approach, which followed the following process:

- identifying the research problem
- identifying relevant studies from empirical and grey literature, archive materials, biblical stories and so on
- collating, summarising and reporting the results to develop the article.

The desk study began with the establishment of a research team having expertise in traditional knowledge, DRR and its concepts. The team provided advice for identifying search terms and databases to address the broad research question. The searches looked at articles in English from electronic databases, using combinations of the following key words: ‘traditional knowledge’, ‘trans-disciplinary’, ‘mitigation’, ‘traditional wisdom’, ‘disaster preparedness’, ‘disaster risk’ and ‘ancient institutions for disaster risk reduction’. The identification of further relevant literature was done by scanning the reference sections of publications in hand. Selection of the databases was done in a way that aimed at making them comprehensive covering issues to address the research problem and objectives.

## Research findings and discussions

### Traditional knowledge in disaster risk reduction – Exploration and acknowledgement

The consulted literature revealed examples of effective traditional DRR measures followed by the indigenous and local communities in different parts of the world. Tables 1 and 2 explore, highlight and acknowledge some of the good practices and achievements of traditional knowledge in DRR.

**TABLE 1:** Traditional knowledge in disaster risk reduction: Good practices from the world around.

Good practices from the world around	DRR strategy	Description of the strategy
1. Early Minoan civilisation of ancient Mediterranean region (Driessen 1987)	Anti-seismic architecture	The building facades of the Minoan civilisation had a different orientation of wall elements which helped to resist the shock from any direction. The absence or near absence of windows in the ground floor of the building helped to sustain the lower floor wall by not weakening it by any opening. The dimension of the room in Minoan houses was much smaller to increase lateral resistance and also to reduce the structural weight of the construction. Lastly, the large houses had a very low number of storeys as houses which are too slender and too high bend more easily during an earthquake.
2. From the 17th-century Xinjiang area of China (Fang et al. 2008)	*Karez*, a traditional irrigation technology to reduce drought disaster.	The basic structure of the *Karez* irrigation was composed of a vertical wall which was dug along the distance of 60 m–100 m in upper reaches, 30 m–60 m in the middle reaches and 10 m–30 m in the lower reaches. A passage was developed to build underground canals by linking numerous vertical wells. To prevent the underground canal from collapsing, wooden pillars were reinforced. When the underground linked canal reached the lower surface, it discharged the water into the small reservoir built on the lower altitudinal surface for the purpose of irrigation. No cost for water uplifting was observed because the topography itself played a vital role in diverting deep water flow, thereby functioning as gravity irrigation. The underground canal also had a minimum impact from evaporation, thus resulting in minimum impact from climate change and thus sustaining the population for thousands of years.
3. From the southern coast of Jamaica in St. Elizabeth (Beckford & Barker 2007).	Drought mitigation strategy	The local people developed a local soil management practice to solve the water scarcity issue. They covered the agricultural field with dried Guinea grass to lessen the soil moisture and to reduce soil erosion on sloping land. Without any involvement of scientific technology, they successfully changed the rainfall deficiency area into a lucrative agro-activity area.
4. From the Aka tribe of the Arunachal Pradesh, India (Namachow, Joshi & Dai 2011).	Cultural beliefs and their mitigation strategies	The Aka tribe considers the mountain *VojoPhu* as sacred. They believed that if anyone extracts any forest material from the sacred mountain, they will lose their way and will bleed to death. Such belief has helped in the conservation of forest resources in and around the mountain area which indirectly has helped in mitigating various natural disasters like floods, drought and landslides.
5. From the Lepcha community of Sikkim Himalaya, India (Jha & Jha 2011).	Folklore and its mitigation strategy	Folklore of the Lepcha community of the Sikkim Himalaya, India, has proved that folklore have developed from the experience and understanding of stories about the natural world around them. According to one folklore tale of the Lepcha community, *Uthis* (Himalayan Alder) jumped from the high cliff when *Guras* (Rhododendron) did not accept her marriage proposal. Thus, Lepcha people believe that in any steep sloping land or landslide prone area the Himalayan Alder grows. This story teaches the listener that in a fresh landslide softwood trees like Himalayan Alder are normally grown and when those trees hold the topsoil then hardwood trees can be grown to convert the landslide prone area into a forested area.

DRR, disaster risk reduction.

**TABLE 2:** Traditional knowledge in disaster risk reduction: Achievements from the world around.

Disaster events	Traditional DRR strategy	Description of the event
1. 17 August 1999, Marmara earthquake in Turkey (Langenbach 2010).	Anti-seismic architecture	Many traditional timber and masonry houses remained standing next to collapsed modern concrete buildings. 25 000 or more people died in the Marmara earthquake, very few of those were trapped in traditionally built infill structures.
2. 26 December 2004, Indian Ocean Tsunami (Syafwina 2014).	Prediction and mitigation	On the island of Simeulue which was just 40 km from the earthquake epicentre which caused the 2004 Indian Ocean tsunami, only seven people were reported killed out of 78 000. The oral tradition consisting of stories and songs carrying the messages of early experience of tsunamis made the local people of Simeulue Island conscious regarding the prediction of tsunamic waves. The oral stories and songs taught people that when an earthquake occurs then they should go to the coast and watch the movement of the tides. If the low tide or retreat of the tide occurs soon after the earthquake, then run towards the higher ground as the low tide will follow up with giant waves.
3. July 2005. Glacial Lake Outburst Flood in Brep village of the Chitral district, Pakistan (Deken 2008).	Early warning and preparedness	The Chitral district barely has 3.5% of suitable agricultural land and consists of steep mountain slopes mostly unsuitable for settlement with a high risk of flash flooding. Because of historical knowledge of flash flooding local people here have gathered the flood adaptation measures by reading the landscape and thus making the interpretation on where to build and not to build their houses. Their historical flood experience has made them understand and interpret early signs of potentially destructive floods too. In the 2005 Glacial Lake Outburst Flood in Brep village of Chitral district, not a single life was lost because the interpretation of stream behaviour acted as the early warning and the village was evacuated in time.
4. 29 December 2009, Tsunami in American Samoa (Rumbach & Foley 2014).	Response strategy	The *Aumaga* (often young and unmarried men who work under the direction of a village chief called Matai) is considered as the ‘hands and feet’ of the village in American Samoa. Being indigenous to the local area they were very fast and familiar with the local environment and were able to respond and rescue victims of the 2009 tsunami before the centralised government or non-governmental institutions arrived.
5. 18 September, 2011 Sikkim Earthquake, India (Khawas & Rai 2017).	Anti-seismic architecture	The Sikkimese traditional structure called *Ekra* house is built of a stone foundation and bamboo wall normally plastered with mud or cement. In spite of being a non-engineered structure, these houses have effectively worked as anti-seismic structures during the 2011 earthquake in Sikkim. Around 40 000 houses were reportedly damaged either fully or partially. Among the fully damaged buildings, about 20% were composed of a Reinforced Cement Concrete (RCC) frame whereas only 4% consisted of traditional construction. According to Arun Rai, a resident of Assamlengy village, East District, Sikkim, India, ‘Ekra house shakes but never falls’.

DRR, disaster risk reduction.

## Recommendations and policy implications

### Need of bridging the gap for scientific research and management: A proposition

As all traditional knowledge cannot be considered useful, recognising the valuable parts is important because this knowledge is on the verge of extinction because of a lack of recognition from the scientific community and because of the increase of commercialisation, urbanisation and subsequent erosion in traditional social networks (Fletcher et al. 2013). Negligence of traditional knowledge in the past and present formal DRR policies is not only because of the predominance of technocratic thinking but also because of the lack of connection between the indigenous people and the non-indigenous external state officials and engineers (Hilhorst et al. 2015).

Traditional methods are normally considered as the most sustainable in nature; however, scientific society argues that these methods lack analytical discourse (Nakashima & Roue 2002) because they are embedded in the culture, traditions, ideology, language and religion of a particular community, and are therefore not universal and difficult to globalise (Maferetlhane 2012). According to researchers, knowledge that lacks universality in its application cannot be considered as knowledge but merely a skill (Nakashima & Roue 2002). A skill here normally refers to the contextual knowledge applicable to and fit for only one local environment. Therefore, the question of broader acceptance or legitimisation always covers the body of traditional knowledge whilst advocating its useful nature in scientific research and management.

However, soon after the 2004 Indian Ocean Tsunami, research in traditional knowledge gained pace among the scientists and policymakers because they were surprised by the traditional prediction system of the Simeulue Island community. These events brought interests towards traditional knowledge in disaster and development research. Modern scientific society and policymakers began to pay attention to how indigenous knowledge could be merged with scientific knowledge to more effectively reduce risk, improve response and recovery, and adapt to long-term climatic change (Rumbach & Foley 2014). All in all documentation of applicable and effective traditional knowledge for DRR becomes very important if the value of traditional wisdom has to merge into scientific research and management. However, merging of useful and effective traditional knowledge into the scientific circle for DRR is not an easy task; therefore, conscious steps need to be taken before integrating traditional wisdom with the conventional methods. This section highlights some of the flexible collaborative steps or approaches required for bridging the gaps between the two knowledge systems in DRR:

- The need to overcome inertia and inflexibility between the two knowledge systems: inertia highlights general resistance to change (Huntington 2000; Nakashima 1993) and inflexibility highlights unwillingness to work together (Huntington 2000; Nakashima 1993). It is normally unacceptable for the traditional knowledge owners to accept and adapt to the new scientific paradigm by leaving their already existing traditional and cultural paradigm (Huntington 2000; Nakashima 1993), and at the same time the inflexibility of modern scientific society in working together with the non-scientist or indigenous (Huntington 2000; Nakashima 1993) or local communities has created a huge gap of isolation between the holders of both knowledge systems. To minimise this gap and to bring in the collaborative approach, it is necessary to first document the effective traditional knowledge and its methods in DRR. This would help to bring up the evidential proof of utility and effective practices of traditional knowledge in DRR, thus paving the way for its integration into scientific methods and practices, thereby slowly helping to overcome the inertia and inflexibility between the two knowledge systems.
- Respecting and trusting each other rather than substituting: the effort must come from both sides. Imposing external knowledge or modern technology of DRR on the indigenous and local people would rather make the road tough for the emergency managers and planners in formulating and implementing the DRR project. It is because native populations basically consider their knowledge to be a part of their own cultural identity (Busingye & Keim 2009). At the same time, relying too much on modern technology alone would make the local people too dependent on external forces (Takeuchi & Shaw 2008) for safeguarding themselves from any disaster. This results in minimising the community’s capacity and ability to help themselves (Takeuchi & Shaw 2008) during natural calamities. Respecting and building up already built-up knowledge of indigenous and local people will empower them to recognise what they can do for themselves (Kelman et al. 2012). By bringing external contributors together with indigenous and local people in collaborative exercise, mutual trust can be fostered through personal connections and thus valuing the strength of different knowledge forms equally (Kelman & Gaillard 2012), as no single knowledge system, either traditional or modern, can be an answer to any sustainability or development activity (Kelman et al. 2012).
- The need for a collaborative DRR exercise between both the knowledge experts: indigenous and local people who have a poor understanding of scientific concept for DRR can discuss with scientists who, in turn, may have a poor understanding of the local context (Kelman et al. 2012). For example, combining the local expertise in a scientific hazard mapping exercise and survey would give a clear verification of the information needed (Deken 2008); therefore, a participatory approach in DRR exercises is significant. The ideal DRR measures should incorporate a balanced mix of modern technology and traditional technology (Takeuchi & Shaw 2008) to make the cost-effectiveness a reality. A good example of collaborative DRR exercise comes from the story of the Amanave village in American Samoa. *Pulenu’u* (village mayor) was selected by the village council to attend the training on how to recognise the warning signs of a potential tsunami event (Rumbach & Foley 2014). During the 2009 American Samoa tsunami, *Pulenu’u* saved all 300 people of the Amanave village by evacuating the entire village in time as soon as the earthquake occurred (Rumbach & Foley 2014).
- The need for capturing effective and applicable traditional wisdom in a systematic way: to bring the successive collaboration approaches or methods between the two knowledge systems in DRR, the knowledge of indigenous and local communities must require a further development in testing, evaluation and refinement (Kelman et al. 2012) in a systematic way. Six steps have been suggested by Syafwina (2014) for the recognition process of effective indigenous knowledge in DRR. The first step gives importance to the assessment process of different traditional knowledge followed by indigenous or local communities. Assessment here refers to the evaluation process of identifying whether the traditional wisdom is useful, valuable or transferable. Therefore, in the second step, its useful function is measured. In the third step, the question of approval or acceptedness of the identified knowledge within the community is raised, and if the knowledge is adopted by the community, then in the fourth step the question or inquiry of its practicality can be raised because indigenous knowledge becomes meaningless without its practical use within its community. In the fifth step, effective practical knowledge in DRR can be transferred through the community, media or family, and in the sixth step the transferred knowledge can be recorded or documented using modern techniques. The effective and practical traditional knowledge tested systematically with trial and error would certainly develop a smooth transition for its integration into scientific methodology for DRR, thus giving birth to a holistic approach.

## Conclusion

All traditional knowledge cannot be treated as a DRR tool unless the local community recognises and uses this knowledge on a daily basis. Without the recognition and utilisation by the local community, indigenous knowledge merely becomes a part of common things in the community (Syafwina 2014). All the effective traditional knowledge that exists has to be documented and captured by using modern techniques. As traditional knowledge holds vast observational data of natural phenomenon, it can help the modern conventional science to understand and analyse natural hazards in more precise ways. Modern technological data alone will not be able to contribute to improve people’s lives unless these are combined with an understanding of local contexts and needs (Deken 2008).

When a certain disaster happens in a particular region, the economically weaker section or transitional group of people are the ones who are mostly affected by the disaster because of the lack of a financial background and sociopolitical status. Economically weaker sections of the society are also more likely to use cheaper construction materials because of lower cost, thus making them vulnerable to earthquake disaster. The costs incurred in the modern disaster reduction process or technologies are normally too high; thus, financial investment for those poor sections of people in reducing the disaster becomes burdensome. Therefore, a complete working balance or integration between traditional technology and scientific technology is necessary to present a viable option in the face of financial concerns for disaster reduction work, because traditional knowledge offers a very cost-effective approach with an environmentally friendly method of DRR. Working together and integrating traditional knowledge with that of external organisations will certainly help legitimise both sets of knowledge system (Kelman et al. 2012).

Traditional knowledge has different sets of ingredients to foster the development of relational or holistic approaches as it involves, interacts with and interconnects humans, non-humans (animals and plants) and nature together, setting a perfect balance for sustainable development and DRR. It is high time that the formal DRR stakeholders should consider and come forward in identifying the age-old knowledge systems and their ingredients for integration into institutional frameworks. Integration is possible if the external stakeholders take the initiative of reconstructing the DRR methodology. Collaboration of the two knowledge experts by encouraging a participatory approach for collecting data and analysing and validating those documented knowledge bases, using a trans-disciplinary approach would definitely pave the way for the creation of hybrid knowledge systems for DRR.
